# Toll-like receptor 4 inhibition sensitizes non-small cell lung cancer to radiotherapy

**DOI:** 10.1080/15384047.2025.2590881

**Published:** 2025-12-03

**Authors:** Ryma Haroun, Cleopatra Rutihinda, Aissatou Hadja Diallo, Juan Pablo Ordonez, Sahar Nassri, Aliya Shams, Maria Fernanda Meza Pacheco, Nour Elhouda Saidi, Lea Bouchard, Guy-Anne Turgeon, Denis Gris, Lee-Hwa Tai, Ayman J. Oweida

**Affiliations:** a Department of Medical Imaging and Radiation Sciences, Faculty of Medicine and Health Sciences, Université of Sherbrooke, Sherbrooke, Quebec, Canada; b Department of Pharmacology and Physiology, Faculty of Medicine and Health Sciences, Université of Sherbrooke, Sherbrooke, Quebec, Canada; c Department of Immunology and Cell Biology, Faculty of Medicine and Health Sciences, Université of Sherbrooke, Sherbrooke, Quebec, Canada

**Keywords:** Radiotherapy, immunotherapy, toll-like receptor 4, HMGB1, radioresistance, non-small cell lung cancer

## Abstract

Non-small-cell lung cancer (NSCLC) is the leading cause of cancer-related mortality worldwide. Although radiotherapy (RT) is used to treat over half of NSCLC patients, about 30% have inherent or acquired radioresistance leading to treatment failure. There's a clinically unmet need to investigate mechanisms of radioresistance that can be targeted in combination with RT. Among these, HMGB1 has been shown to play a key role in tumor progression. Our research investigates TLR4, a receptor for HMGB1, highly expressed in NSCLC tissues, as a mediator of radioresistance.

**Methods:**

The TLR4 inhibitor, TAK242, was tested in NSCLC cell lines (murine: LLCI, KLN205; human: H1975, SW900). Cells were irradiated at 2 and 10 Gy. *In vivo*, KLN205 cells were implanted in DBA/2 mice and tumors were irradiated at 10Gy. Gene and protein expression of TLR4 and MyD88 were assessed *in vitro* and *in vivo*. HMGB1 secretion was quantified after RT. Clonogenic assays were performed to evaluate the effect of TAK242 on radiosensitivity *in vitro*. The combination of TAK242 and RT was investigated *in vivo* in mice bearing KLN205 tumors.

**Results:**

TAK242 significantly decreased NSCLC cell proliferation and migration. Radiation at 2 and 10 Gy increased TLR4 gene expression *in vitro* and *in vivo* in a dose-dependent manner. *In vitro*, TLR4 and HMGB1 protein expression was upregulated following radiation. TAK242 in combination with radiation enhanced radiosensitivity *in vitro*. TAK242 decreased the percentage of cells in the G1 phase, coupled with an increase in late S and G2/M, suggesting radiosensitization via cell cycle modulation. *In vivo*, the combination of RT and TAK242 significantly reduced growth of KLN205 tumors.

**Conclusion:**

These findings show that TLR4 inhibition enhances RT sensitivity in NSCLC.

## 1 Introduction

Over 50% of NSCLC patients undergo radiotherapy (RT) treatment, underscoring its widespread utilization in managing the disease.^1^^,^^2^ Early-stage NSCLC patients are treated with stereotactic ablative body radiotherapy (SABR) consisting of high-dose RT per fraction (10−18 Gy) over 3−5 d.^3^ Single-day SABR treatment is also becoming common with a single fraction of 30−34 Gy.^4^ In stage II−III NSCLC disease with lymph node involvement, the dose delivered each day must be decreased to spare adjacent organs at risk, leading to the use of hypofractionation (3‒4 Gy per fraction) or standard fractionation regimens (2 Gy per fraction) up to 60−66 Gy over 3−6 weeks.^5^ Despite advancements in RT delivery techniques and variations in dose and fractionation, 30%−50% of NSCLC patients experience locoregional treatment failure**.**^6-8^ For NSCLC patients who fail RT, curative treatment options are limited. Variables such as radiation dose, gross tumor volume, and fraction size can significantly affect local control.^9^ In addition, intrinsic tumor radioresistance is a major impediment to successful RT treatment.^10^ Tumors can be intrinsically radioresistant or, can acquire features in response to radiation that render them radioresistant**.**^11^^,^^12^ There is a dire need to elucidate the factors underlying heterogeneous treatment responses and why certain tumors exhibit excellent responses to RT, while others are radioresistant.

TLR4 is among the most well-studied Toll-like receptors (TLRs). TLR4 expression has been observed in various cancer cells and implicated in promoting tumor cell survival, proliferation, and metastasis.^13^ Endogenous ligands such as HMGB1, which are released either passively from damaged necrotic cells or actively from immune and/or stressed cells,^14^ act as damage-associated molecular patterns (DAMPs)^15^ and induce the activation of TLR4.^14^ Upon activation, TLR4 recruits MyD88 to its cytoplasmic domain. MyD88 serves as an adapter protein, initiating a cascade of downstream signaling events.^16^ This leads to the activation of the NF-κB and MAPK pathways and a variety of proinflammatory cytokines, including interleukins such as IL-1, IL-6, and IL-8.^17^ It has been demonstrated that these molecules, especially IL-6, are capable of inducing radioresistance by activating the STAT3 signaling pathway and EMT, enhancing DNA repair, and suppressing radiation-induced oxidative stress and apoptosis, thereby promoting the survival of cancer cells in a plethora of cancers, including NSCLC.^11^^,^^18^^,^^19^

Among TLR4 inhibitors, TAK-242 (also known as resatorvid) is a highly specific small molecule inhibitor.^20^ TAK-242 has shown anticancer effects in several cancers. *In vitro*, treatment of ovarian cancer cells with TAK-242 alone or in combination with chemotherapy decreased cancer cell survival and invasion, induced cell cycle arrest, and reduced the expression of IL6.^21^ Another study showed the same effect in breast cancer.^22^ Zandi et al showed that TAK-242 reduces the enzymatic activity of MMP2 and MMP9 and downregulates the expression of epithelial-mesenchymal transition (EMT)-related genes in ovarian and breast cancer cells.^23^

To the best of our knowledge, no study has investigated the effect of radiation on TLR4 expression in NSCLC, nor has any study attempted to combine TAK242 with radiation. This gap in the literature is significant, given the well-documented role of TLR4 in mediating tumor progression. RT is known to induce the release of DAMPs such as HMGB1, which are key activators of the TLR4 signaling pathway. Understanding how radiation influences TLR4 expression is critical for deciphering the complex interplay between RT and the tumor microenvironment (TME), particularly in the context of inflammatory signaling.

Furthermore, the potential of TAK242 to inhibit TLR4 activation and mitigate radiation-induced protumorigenic effects represents a promising avenue to enhance the therapeutic efficacy of RT. By combining TAK242 with radiation, we aimed to explore whether targeting TLR4 can improve radiosensitivity, reduce tumor growth, and counteract the mechanisms of radioresistance. This approach could pave the way for novel combination therapies that exploit the dual benefits of TLR4 inhibition and RT to achieve better clinical outcomes for NSCLC patients.

## 2 Results

### 2.1 TLR4 inhibition induces apoptosis and reduces NSCLC cell proliferation

**Figure 1.**
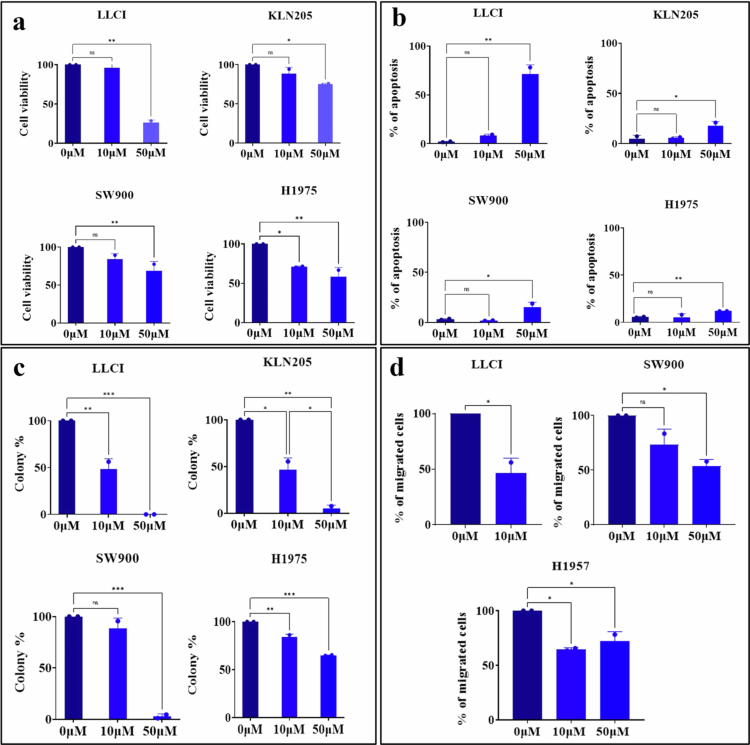
TAK242 inhibits the proliferation and migration of NSCLC cells. (a) LLCI and KLN205 cells were treated with 10 or 50 µM TAK242 for 48 h, and cell viability was assessed by MTT assay. (b) Apoptosis was measured by Annexin V/PI staining after 24 h of treatment. (c) Colony-forming ability was evaluated after 48 h of treatment. (d) Cell migration was quantified following 24 h of treatment. Data are presented as mean ± SD from three independent experiments. Statistical analysis was performed using one-way ANOVA with Dunnett’s post-test. Significant differences versus untreated controls are indicated as *P < 0.05, **P < 0.01, ***P < 0.001.

The NSCLC cell lines KLN205, LLCI, H1975, and SW900 were treated with TAK242 at concentrations of 0, 10, and 50 µM for 48 h to evaluate its impact on cancer cell proliferation. At 50 µM TAK242, the LLCI cell line demonstrated the highest sensitivity, with a marked reduction of over 70% in cell viability. KLN205 and SW900 also showed statistically significant decreases in viability at this concentration, with reductions of 25% and 32%, respectively, compared to DMSO-treated controls (Figure 1a). At the lower concentration of 10 µM, only the H1975 cell line exhibited a notable 30% decrease in viability, whereas the reductions in the LLCI, KLN205, and SW900 cell lines did not reach statistical significance (Figure 1a).

We then evaluated the effect of TAK242 treatment on apoptosis using Annexin V and PI staining. Consistent with the MTT assay results, TAK242 treatment significantly increased apoptosis in all cell lines at a concentration of 50 µM (Figure 1b). LLCI showed the greatest sensitivity with 71% of cells showing apoptosis compared to the control. The KLN205, SW900, and H1975 cell lines showed less sensitivity with percentages of 17.5%, 15.3%, and 11.8%, respectively.

To investigate the effect of TAK242 on colony formation, we analyzed the number of colonies formed by different cell lines in the presence or absence of the treatment. LLCI and KLN205 exhibited a similar response to TAK242 (Figure 1c). LLCI showed a 52% reduction in the percentage of colonies formed after treatment with 10 µM, and no colonies were formed with 50 µM of treatment. Similarly, KLN205 showed a 53.5% decrease in the percentage of colonies at 10 µM and 95% with 50 µM treatment. For the SW900 cell line, treatment at 10 µM did not show a significant response, while treatment at 50 µM significantly reduced the percentage of colonies by 97.3%. H1975 cell line showed significant results with both concentrations of TAK-242, with a 16.5% reduction with 10 µM treatment and 35% with 50 µM concentration.

Taken together, our results show that TAK-242 is a potent inhibitor of cell proliferation and colony formation in murine and human NSCLC cell lines.

### 2.2 TAK242 reduces migration of NSCLC cells

Given the potent cytotoxic effects of TAK-242 on cell proliferation, we investigated its effect on cell migration using the Transwell migration assay. Our results showed that TAK-242 significantly decreased the migration of LLCI, H1975, and SW900 cells compared to DMSO-treated controls (Figure 1d). LLCI was the most sensitive among the four cell lines with nearly 46% reduction in the percentage of migrated cells after 24 h of 10 µM TAK242 treatment, compared to control. The 50  µM dose was excluded for LLCI cells, as it induced 71% cytotoxicity (Figure 1b). H1975 cells showed a 64.5% reduction in cell migration with 10 µM TAK-242. SW900 cells showed a 27% reduction at the same concentration, which was not statistically significant. A significant reduction of 53.8% in SW900 cell migration was observed with 50 µM TAK-242.

### 2.3 Radiation induces expression of TLR4 and MyD88 *in vitro* and *in vivo* in NSCLC

**Figure 2.**
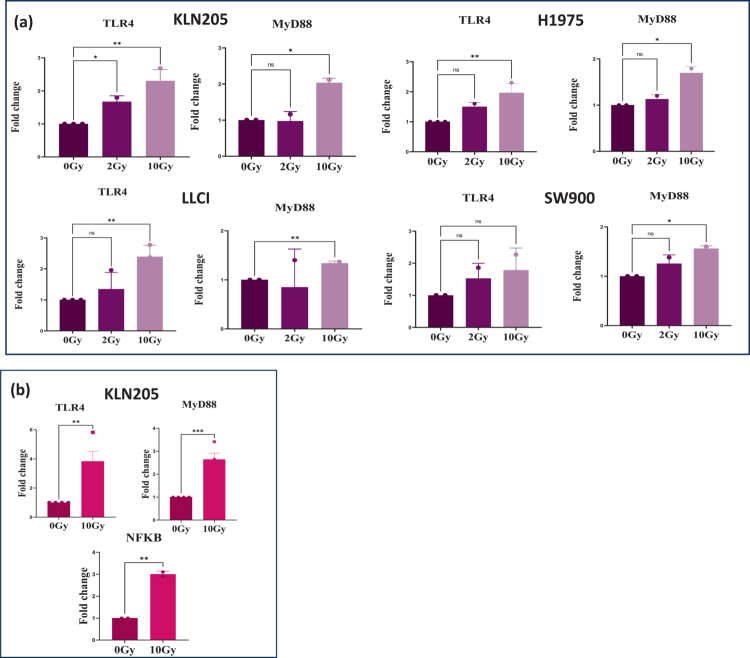
Radiation induces the expression of TLR4 and MyD88 *in vitro* and *in vivo*. (a) NSCLC cells were irradiated at 2 and 10 Gy. Gene expression was measured 24 h post radiation to investigate the impact of radiation in TLR4 and MyD88 expression. qPCR results showed that TLR4/MyD88 gene expression increases with exposure to 2 Gy and further 10 Gy RT *in vitro*. (b) KLN205 cells were subcutaneously injected into DBA/2 mice. Tumors were irradiated at a dose of 10 Gy. The expression of TLR4, MyD88, and NFКB was measured *in vivo*. Data are shown as mean ± SD from three independent experiments. Statistical analysis was performed using ordinary one-way ANOVA followed by Dunnett's multiple comparisons test. Statistically significant differences compared to the untreated control group are indicated as *P < 0.05, **P < 0.01, ***P < 0.001.

Increasing evidence indicates that TLR4 expression plays a pivotal role in cancer progression and treatment resistance. Given that radiation induces the release of HMGB1, a known ligand of TLR4, we suggest that radiation might activate TLR4 signaling through HMGB1, potentially promoting radioresistance. To investigate this, we used our panel of murine and human NSCLC cell lines (LLCI KLN205 SW900 and H1975). The cells were treated with sham-control or irradiated with 2 Gy or 10 Gy. Our results showed a 1.6−2.2-fold increase in TLR4 gene expression across the four cell lines after 10 Gy radiation relative to control (Figure 2a). No significant difference was observed after 2 Gy radiation. We also analyzed downstream expression of MyD88 and observed a similar increase of 1.3−2.1-fold after 10 Gy but not 2 Gy relative to control (Figure 2a). For the *in vivo* mouse model, KLN205 cells were injected subcutaneously into the right flank of DBA/2 mice, and tumors were subjected to 10 Gy irradiation when they reached 200 mm³. Tumors were subsequently collected for RNA extraction 24 h post-RT. Irradiated KLN205 tumors showed elevated gene expression for TLR4 (3.8-fold increase), MYD88 (2.6-fold increase) and NFКB (3-fold increase) compared to 0Gy (Figure 2b). Taken together, our *in vitro* and *in vivo* data suggest that RT increases TLR4 expression in NSCLC.

### 2.4 Radiation enhances HMGB1 secretion and upregulates TLR4 expression in NSCLC cell lines

**Figure 3.**
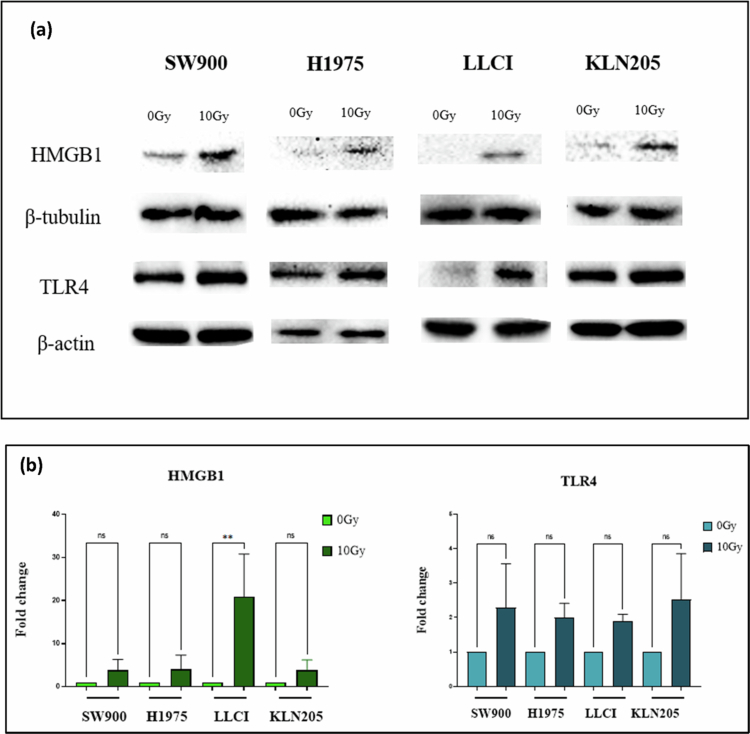
Radiation induces TLR4 protein expression and the release of HMGB1 in conditioned media. NSCLC cells were irradiated at 10 Gy. Protein expression was measured 24 h post radiation to investigate the impact of radiation on TLR4 expression. (a) Western blot results showed that TLR4 protein expression increases with 10 Gy exposure *in vitro*. Radiation induces the release of HMGB1 in the conditioned media of the irradiated cells. (b) Western blot results were quantified and normalized to *β*-actin and *β*-tubulin for TLR4 and HMGB1 protein expression, respectively. Data are shown as mean ± SD from two independent experiments. Statistical analysis was performed using ordinary one-way ANOVA followed by Dunnett's multiple comparisons test (GraphPad Prism). Statistically significant differences compared to the untreated control group are indicated as *P < 0.05, **P < 0.01, ***P < 0.001.

To investigate how radiation induces TLR4 activation, NSCLC cells were irradiated with 10 Gy, and proteins were collected 24 h post-irradiation from both cell lysates and conditioned media. Western blot analysis was conducted to assess TLR4 expression in cell lysates and HMGB1 secretion in conditioned media (Figure 3a).

HMGB1 levels increased in the conditioned media of irradiated cells compared to non-irradiated controls. The secretion of HMGB1 was elevated by 3−4-fold in KLN205, SW900, and H1975 cells, and by 20-fold in LLCI cells.

In parallel, TLR4 protein expression was elevated in the lysates of irradiated cells, with fold changes ranging from 1.4 to 2.0 across the different cell lines (Figure 3b). These results demonstrate that radiation promotes both the secretion of HMGB1 and the upregulation of TLR4 expression. Together, these findings support the hypothesis that HMGB1 released in response to radiation activates TLR4, potentially contributing to radiation-induced signaling mechanisms in NSCLC cells.

### 2.5 Combination treatment of TAK242 and radiation in NSCLC cells leads to reduced clonogenic cell survival

To investigate whether TLR4 inhibition using TAK242 can radiosensitize NSCLC cell lines *in vitro*, we performed the gold-standard clonogenic assay. NSCLC cells were treated with TAK242 or DMSO for 4 h prior to irradiation. Cells were then irradiated with increasing doses of radiation (2, 4, 6, and 8 Gy). We used 10 µM TAK242 based on the MTT assay results shown in Figure 1, which revealed that this concentration has a minimal effect on cell toxicity. In addition, we used 25 µM TAK242 to verify the effect of a higher dose of TAK242 in radiosensitizing cells without reaching the IC_50_ of TAK242.

**Figure 4.**
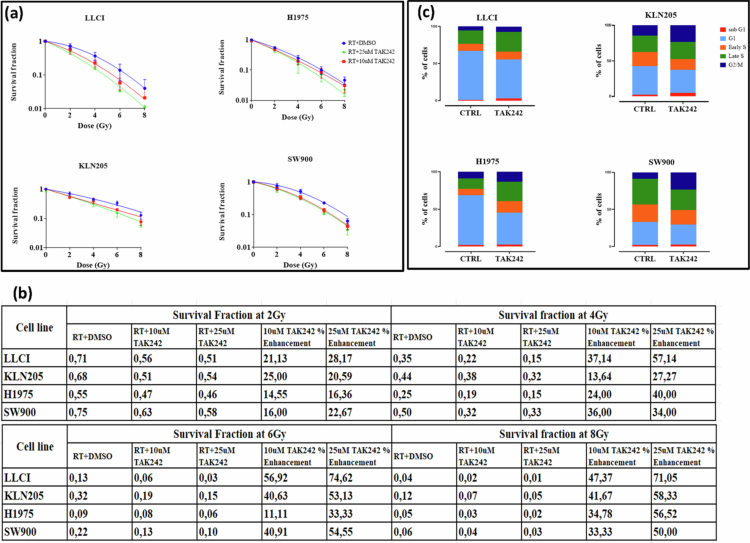
Clonogenic assay for radiosensitivity. Clonogenic survival of NSCLC cell lines after exposure to DMSO and TAK242. (a) Data shows the mean survival fraction of three independent experiments. Error bars represent standard deviation. Connecting lines show the linear-quadratic fit of the data points. Two-tailed t-test was performed to assess significance at each data point between the groups. (b) Table summarizing the survival fractions at 2 Gy, 4 Gy, 6 Gy and 8 Gy for RT + DMSO and RT + TAK242. The percentage increase represents the percentage decrease in SF in RT + TAK242 relative to RT + DMSO. (c) TAK242 Induces Cell Cycle Redistribution. NSCLC cells treated with 25 µM TAK242 for 48 h exhibited alteration in cell cycle distribution, as analyzed using the Click EDU kit. Data represent the mean of two independent experiments.

The combination of TAK242 and radiation significantly decreased clonogenic survival across all tested cell lines. Analysis of the survival fraction at 2 Gy (SF2) demonstrated that H1975 cells exhibited greater intrinsic radiosensitivity (SF2 of 0.55) compared to KLN205, LLCI, and SW900 (SF2 values of 0.75, 0.71, and 0.68, respectively) (Figure 4a). The most substantial reduction in clonogenic survival between the radiation + DMSO and radiation + TAK242 treatments occurred at 6 Gy with 25 µM TAK242, resulting in more than a 50% decrease in clonogenic survival for all cell lines, except H1975, which showed a reduced sensitivity, with only a 30% reduction in survival fraction. LLCI cells exhibited the most pronounced response, with over 70% reduction in clonogenic survival following the combination of 25 µM TAK242 and 6 Gy or 8 Gy radiation. The percentage enhancement in survival reduction for the combined treatments is illustrated in Figure 4b.

### 2.6 TAK242 induces cell cycle redistribution and radiosensitization in NSCLC cells

Given the significant effect of TLR4 inhibition on clonogenic survival of NSCLC cells, we performed cell cycle analysis to determine whether TAK242 enhances radiosensitization through cell cycle modulation. Our analysis of the cell cycle distribution following 48 h of TAK242 treatment revealed alterations across multiple cancer cell lines (Figure 4c). In KLN205, LLCI, H1975, and SW900 cells, we observed a consistent decrease in the proportion of cells in the G1 phase. This reduction in G1 phase was accompanied by a concomitant increase in the percentage of cells in the late S phase and G2/M phase. Notably, in LLCI and KLN205 cell lines, we also detected an increase in the sub-G1 population, which is often indicative of apoptotic cells. These cell cycle perturbations, particularly the decrease in G1 phase cells and the accumulation in late S and G2/M phases, are consistent with patterns associated with enhanced radiosensitivity. The increase in sub-G1 population in LLCI and KLN205 cells further suggests that TAK242 may be inducing cell death in these lines. These findings collectively indicate that TAK242 treatment induces cell cycle changes that are generally associated with increased cellular vulnerability to radiation. The observed shift from G1 to late S and G2/M phases, where cells are typically more radiosensitive, suggests that TAK242 may be priming these cancer cells for enhanced response to RT.

### 2.7 Treatment with TAK242 enhances tumor response to radiotherapy

To investigate the effect of TLR4 inhibition using TAK242 in radiosensitizing NSCLC *in vivo*, we injected KLN205 cells into the right flank of DBA/2 mice. When tumors reached 200 mm^3^, the mice received two intraperitoneal injections of 10 mg/kg of TAK242. The tumors were exposed to 10 Gy dose of radiation after the two first injections of TAK242. Mice received injections of TAK242 twice per week, for a duration of 21 d. The control mice received an intraperitoneal injection of DMSO. Tumor volume was monitored using calipers.

Tumor volume measurements demonstrated that the combination of TAK242 and RT had the most pronounced effect on reducing tumor growth. Tumors in the control and TAK242-only groups showed a steady increase in volume, with no significant differences between these groups. In contrast, RT alone resulted in a moderate reduction in tumor growth, reaching its minimum value on day 17, before resuming growth thereafter, reducing tumor volume by approximately 23% compared to the control.

**Figure 5.**
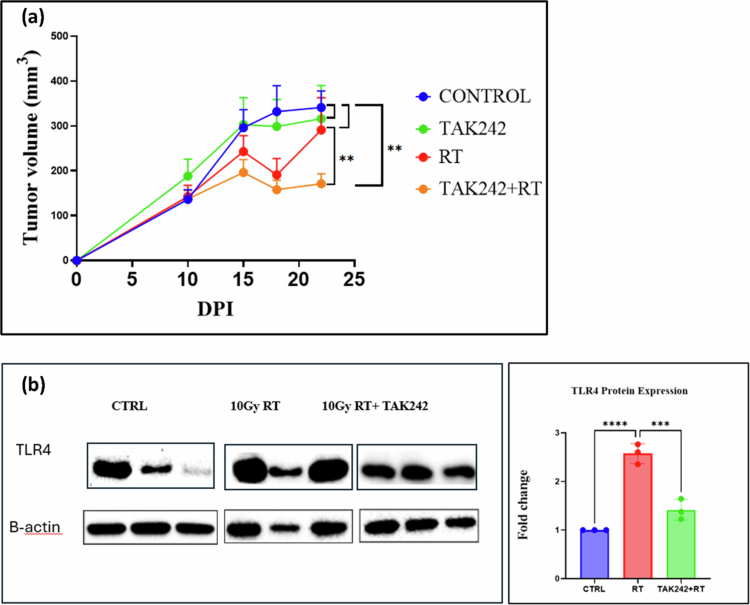
Effect of TLR4 Inhibition with TAK242 on radiosensitizing NSCLC *in vivo*. (a) KLN205 cells were injected into the right flank of DBA/2 mice to develop tumors. Upon reaching a volume of 200 mm², mice received two intraperitoneal injections of 10 mg/kg TAK242 prior to a 10 Gy radiation dose. TAK242 injections continued twice weekly for 21 d. Tumor volumes were measured with calipers three times per week and are presented as mean ± SD. Statistical analysis was performed using two-way ANOVA with repeated measures (GraphPad Prism), followed by Tukey's multiple comparisons test to evaluate differences between treatment groups. (b) Protein lysates were prepared from excised tumors following the treatment course and analyzed by Western blot for TLR4 expression. *β*-actin was used as a loading control. Statistical analysis was performed using one-way ANOVA followed by Tukey's multiple comparisons test to compare control vs 10 Gy RT and 10 Gy RT vs 10 Gy RT + TAK242.

Notably, the combination of TAK242 and RT significantly suppressed tumor growth compared to both RT alone and TAK242 alone, as evidenced by the tumor volume at 25 DPI. The mean tumor volume in the TAK242 + RT group was reduced to approximately 160 mm³, 260 mm³ in the RT-only group and 340 mm³ in the control and TAK242-only groups. The combination therapy resulted in a nearly 36% reduction in tumor volume compared to RT alone and a 50% reduction compared to the control group. These findings suggest a synergistic interaction between TAK242 and RT, enhancing the overall therapeutic efficacy in reducing tumor progression (Figure 5a).

To validate that TAK242 effectively inhibits TLR4 *in vivo* and that this inhibition contributes to radiosensitization, we analyzed TLR4 protein levels in excised tumors by Western blot. We found that RT alone led to an upregulation of TLR4 protein expression. In contrast, tumors from mice treated with TAK242 in combination with RT showed a marked reduction in TLR4 protein levels compared to RT, confirming that TAK242 effectively suppresses TLR4 in the tumor. These findings indicate that the enhanced tumor growth suppression observed with the combination therapy is associated with TLR4 inhibition, supporting a mechanistic link between TAK242-mediated TLR4 suppression and radiosensitization (Figure 5b).

## 3 Discussion

Considering the elevated expression of TLR4 in lung cancer and its role in cancer cell proliferation, we investigated the impact of TLR4 inhibition on the proliferation of NSCLC cell lines. Given the significant heterogeneity of NSCLC, we selected a panel of diverse cell lines to represent this complexity within NSCLC. The cell lines used in this study (LLCI, KLN205, H1975, and SW900) differ notably in their mutational profiles and responses to therapies. LLCI and KLN205 are of murine origin, whereas H1975 and SW900 are derived from human tumors. H1975 is a human lung adenocarcinoma cell line, characterized by L858R and T790M mutations in the EGFR gene, which confer resistance to EGFR tyrosine kinase inhibitors and a distinct tumorigenic and treatment-resistant behavior.^24,^^25^ SW900 represents human lung squamous cell carcinoma and carries molecular alterations such as TP53 mutations, known to influence tumor aggressiveness and resistance to apoptosis.^26^ LLCI, widely employed in animal models to study tumor progression and invasion, harbors mutations including K-ras that affect its oncogenic properties.^27^ KLN205, another murine squamous cell carcinoma line, is used for its invasive characteristics and resistance to specific therapeutics.^28^ The selection of these biologically and molecularly distinct cell lines enhances the relevance of our findings by more accurately reflecting the diverse nature of real tumors.

Our study demonstrated that inhibiting TLR4 using TAK242 induced significant cytotoxicity across all tested NSCLC cell lines (LLCI, KLN205, SW900, and H1975). This finding aligns with previous research showing similar cytotoxic effects of TAK-242 in ovarian and breast cancer cells, indicating a broader applicability of TLR4 inhibition as a therapeutic strategy.^23^

Cell migration is a key step in metastasis, allowing cancer cells to invade adjacent tissues and disseminate to distant organs. By inhibiting cell migration, TAK242 can directly interfere with the metastatic cascade, reducing the likelihood of secondary tumor formation. Our results are consistent with prior studies linking TLR4 activation to enhanced migratory and invasive behaviors in various cancer types. For instance, TLR4-mediated signaling has been shown to promote EMT, a process that endows cancer cells with increased motility and resistance to apoptosis.^29^ TAK242's ability to disrupt these pathways further underscores its potential as a therapeutic agent targeting not only primary tumor growth but also metastatic dissemination. Similarly, colony formation assays are indicative of the clonogenic potential of cancer cells, which reflects their ability to survive, proliferate, and establish new tumor colonies. The reduction in colony formation observed in our study suggests that TAK242 not only hampers the proliferative capacity of NSCLC cells but also limits their ability to withstand microenvironmental stressors that typically favor tumor survival and growth.

Given that RT induces DNA damage and causes release of DAMPS,^30^ we assessed the secretion of HMGB1, a key ligand for TLR4, after RT. Our results showed that RT induces the secretion of HMGB1 by NSCLC cells. These results have been validated in other cancer cell lines. A similar study by Dong et al., highlighted the role of HMGB1 in contributing to radioresistance in esophageal squamous cell carcinoma cells overexpressing HMGB1.^30^ HMGB1-overexpressing cells had significantly higher cell proliferation rates than negative control groups with or without irradiation. The group demonstrated that HMGB1 expression correlates with *γ*-H2AX expression, a key marker of DNA damage in tumor cells after RT, highlighting the interplay between HMGB1 and RT-induced DNA damage in cancer cells. Another study showed that extracellular HMGB1 initiates signaling through TLR4, leading to NF-κB phosphorylation.^31^ This cascade subsequently induced the transcription of Snail and Twist genes, pivotal regulators of EMT, and activated MMP2, an enzyme involved in extracellular matrix remodeling. These molecular events collectively contribute to altering the intrinsic characteristics of tumors, promoting their invasive potential and metastatic behavior**.**

Our study further investigated the effect of radiation on the activation of the TLR4 signaling pathway. Our results showed that radiation significantly increased the expression of TLR4 and MYD88 *in vitro* and in KLN205 tumors *in vivo*. Additionally, we observed a marked increase in NF-κB expression following RT *in vivo*. These findings suggest that RT not only induces DNA damage and DAMP release but also actively engages the TLR4 signaling pathway, potentially contributing to a protumorigenic microenvironment.

The upregulation of TLR4 and MYD88 in response to radiation is particularly significant, as these molecules are central components of the TLR4 signaling cascade. MYD88 serves as a key adapter protein, linking TLR4 activation to downstream signaling events, including NF-κB activation. The observed increase in NF-κB expression aligns with its role as a master regulator of inflammation, cell survival, and immune evasion. In the context of cancer, NF-κB activation can promote tumor progression by enhancing the transcription of genes involved in cell proliferation, antiapoptotic pathways, angiogenesis, and metastasis.

Finally, we investigated the effect of TLR4 inhibition on the radiosensitivity of NSCLC *in vitro* and *in vivo*. Our results showed that treatment with TAK242 induces the sensitization of NSCLC cells to radiation *in vitro*. Our cell cycle analysis revealed that TAK242 treatment induces significant alterations in cell cycle distribution across multiple NSCLC cell lines, complementing our previous findings on apoptosis induction and radiosensitization. The observed decrease in cells in the G1 phase, coupled with an increase in late S and G2/M populations, aligns with patterns associated with enhanced radiosensitivity in cancer cells. These findings are consistent with studies by Pawlik and Keyomarsi,^32^ who demonstrated that cells in late S and G2/M phases are generally more radiosensitive than those in G1 or early S phase. Our results suggest that TAK242 may be priming NSCLC cells for enhanced radiation response by shifting them into these more vulnerable phases of the cell cycle. Interestingly, our observation of an increased sub-G1 population in LLCI and KLN205 cells parallels findings by Lin et al.,^33^ who reported that TLR4 inhibition in lung cancer cells led to increased apoptosis and cell cycle arrest. This suggests that TAK242's effects on cell cycle and apoptosis may be mediated, at least in part, through its action on TLR4 signaling. The G2/M accumulation we observed is particularly noteworthy, as it is reminiscent of the radiosensitizing effects of other agents, such as paclitaxel, which has been shown to enhance radiotherapy efficacy through G2/M arrest.^34^ This suggests that TAK242 might be leveraging similar mechanisms to enhance radiosensitivity. Moreover, the reduction in G1 phase cells could indicate impaired DNA repair capacity, as suggested by Branzei and Foiani,^35^ who highlighted the importance of the G1 phase for certain DNA repair processes. This could further contribute to TAK242's radiosensitizing effects by limiting the cells' ability to repair radiation-induced damage. Our *in vivo* results showed that TAK242 in combination with RT reduced tumor growth significantly compared to RT alone.

**Figure 6.**
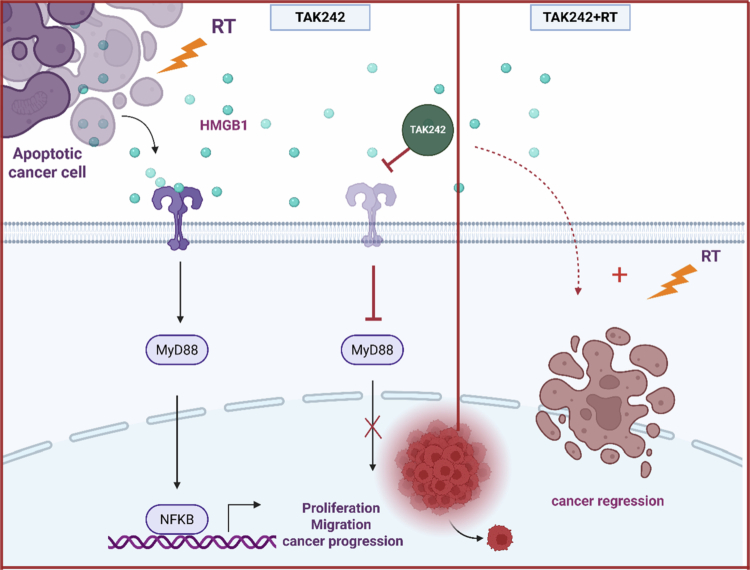
RT induces the release of HMGB1 from apoptotic cancer cells, which activates its receptor TLR4. This activation triggers the downstream TLR4/MYD88/NF-κB signaling cascade, leading to increased cancer cell proliferation, migration, and radioresistance, thereby contributing to tumor progression. Inhibition of TLR4 with TAK242 prevents NF-κB activation and suppresses tumor-promoting effects. The combination of TAK242 with RT results in tumor regression and enhanced therapeutic efficacy.

To our knowledge, this is the first study to demonstrate that RT activates the TLR4 signaling pathway in NSCLC. This novel finding has significant implications. While RT is a cornerstone of NSCLC treatment due to its ability to induce DNA damage and cell death, our results suggest that it may inadvertently activate pathways that support tumor survival and progression. Based on our data, we propose a model (Figure 6) in which RT activates TLR4 signaling, thereby promoting tumor cell survival and radioresistance. Conversely, TLR4 inhibition with TAK-242 enhances radiosensitivity both *in vitro* and *in vivo*, suggesting a promising therapeutic strategy that may become clinically viable in the near future and improve outcomes in NSCLC patients.

## 4 Materials and methods

### 4.1 Cell culture

The human NSCLC cell lines, H1975 and SW900 and the murine cell line, LLCI were obtained from the lab of Dr Lynn Heasley (University of Colorado). KLN205 was obtained from the ATCC (Manassas, VA). The cells were maintained at 37 °C and 5% CO2 in a humidified incubator. H1975 and SW900 cells were cultured in RPMI media supplemented with 10% fetal bovine serum (FBS), 4 mM L-glutamine, 4500 mg/L glucose, 1 mM Na Pyr and 1500 mg/L Na bicarbonate. Murine LLCI and KLN205 cells were cultured in DMEM and EMEM, respectively. DMEM was supplemented with 10% FBS, 4 mM L-glutamine, 4500 mg/L glucose, 1 mM Na Pyr and 1500 mg/L Na bicarbonate. EMEM was supplemented with 20% FBS, 1% Pen-strep, 1 mM sodium pyruvate, 2 mM L-glutamine and 0.1 mM NEA

### 4.2 qPCR

RNeasy Mini Kit (Qiagen, Toronto, ON) was used to extract total RNA from cells according to the manufacturer's protocol. The RNA concentration and quality were determined with BioDrop™μLITE (Montreal Biotech, Montréal, QC). The first complementary strand was made from 1 μg total RNA using the Superscript Reverse Transcription kit (Life Technologies Inc., Burlington, ON) according to manufacturer's protocol. TaqMan™ Fast Advanced Master Mix (Life Technologies Inc, Burlington, ON) was used to amplify and detect the DNA products. Gene expression analysis was performed using the QuantStudio™ 3 Real-Time PCR System (ThermoFisher Scientific, Montréal, QC). TLR4, MyD88, NFКB and GAPDH were probed using TaqMan assays (Life Technologies Inc., Burlington, ON).

GAPDH was used as the internal reference for other genes. All primers showed more than 90% efficiency with a single melting curve. Expression levels of the housekeeping gene (GAPDH) were used to calculate fold induction of the specific genes modulated by RT. Ct values of the genes were used to determine the relative fold change through the 2−2−ΔΔCt method.

### 4.3 Western blot

NSCLC cells were irradiated with a single dose of 10 Gy. After 24 h, the cells were lysed with RIPA buffer (Life Technologies, Burlington, ON), containing phosphatase and protease inhibitors. Protein concentrations were determined using BCA Protein Assay kit (ThermoFisher, Montreal, QC).

Conditioned media from the same samples were collected and centrifuged. Proteins in the conditioned media were quantified using BCA Protein Assay kit.

Equal amounts of protein (30 µg of whole-cell lysates) were loaded and separated for 1 h at 200 V on SDS–polyacrylamide gels (10%) and transferred to PVDF membranes overnight at 30 V (Bio-Rad, Montreal, QC). The membranes were blocked with 5% nonfat milk in TBST and incubated overnight at 4 °C with gentle agitation with primary antibodies against TLR4 (Sigma, Montreal, QC; 1:1000 dilution), HMGB1(Abcam, Cambridge, Cambs; 1:1000 dilution). Antirabbit and Antimouse secondary antibodies (Millipore-Sigma Inc, Oakville, ON; 1:5000 dilution) were added to the respective membranes for 1 h at room temperature. *β*-actin and *β*- tubulin (cell signaling, Montreal, QC; 1:5000 dilution) were used as a loading controls. The signals were visualized using enhanced chemiluminescence (ECL) reagents (Bio-Rad, Montreal, QC), detected using ChemiDoc (Bio-Rad, Montreal, QC) and analyzed using Image Lab software.

For *in vivo* experiments, KLN205 tumors were excised from the mice after five consecutive daily intraperitoneal injections of TAK242(10 mg/kg) and four days postirradiation (10 Gy, given after the first dose of TAK242) to capture both the effects of radiation and TAK242. Tumors were snap-frozen and homogenized in RIPA buffer containing phosphatase and protease inhibitors. The protein concentration was determined using the BCA protein assay kit. Equal amounts of protein (30 µg) were loaded, and the membranes were immunoblotted with TLR4 and *β*-actin antibodies; all other procedures, including detection and quantification, were performed as described above for *in vitro* samples.

### 4.4 Irradiation

For *in vitro* irradiation, the cells were irradiated at room temperature using a Gamma Cell 3000 irradiator. Doses of 2 or 10 Gy were given as a single dose at a dose rate of 9.18 Gy/min. For *in vivo* irradiation of mouse tumors, external beam gamma radiation was performed by the Leskell Gamma knife (GK) (Elekta Instruments AB). Briefly, treatment planning was performed using 4-mm collimators delivered at predetermined coordinates targeting the tumor. A radiation dose of 10 Gy was applied to the tumor located on the right flank. The mice were anesthetized using 1.5 L/min oxygen containing isoflurane and positioned on a custom-designed stereotactic frame that is compatible with the couch of the GK.

### 4.5 MTT proliferation assay

LLCI, KLN205, H1975, and SW900 cells were seeded in 96-well plates in triplicate at a density of 600 cells/well for LLCI and 2000 cells/well for the rest of the cell lines, at 37 °C and 5% CO2 for 24 h. TAK242 treatment or PBS for control was added for 48 h. Cell viability assay was performed using the methylthiazol tetrazolium (MTT) for 4 h in the incubator. DMSO was added for 30 mn. Absorbance was measured using a microplate reader at 595 nm wavelength.

### 4.6 Transwell migration assay

Transwell migration assays were performed using migration chambers with 8.0-*µ*m-pore polycarbonate membranes (Sarstedt, Montreal, QC). 3 × 10^4^ cells/well was seeded for LLCI cell line and 4 × 10^4^ cells/well for KLN205, H1975 and SW900 cell lines in 100 *µ*l of serum-free medium in the upper chambers, and 500 *µ*l of medium supplemented with 10% FBS was added to the lower chambers. After 5 h of incubation, the cells were treated with 10 and 50 µM TAK242. After 24 h of treatment, cells on the upper surface were washed with PBS and removed with a cotton swab. The lower surface of the membrane was fixed with 10% formalin for 15 min and stained with 0.5% crystal violet solution for 30 min. The membranes were removed, placed in a 96-well plate and crystal violet staining was eluted using 30% acetic acid. The absorbance was measured using a microplate reader at a wavelength of 595 nm.

### 4.7 Flow cytometric analysis of apoptosis

To assess the induction of apoptosis, flow cytometry analysis was performed. LLCI (1 × 10^4^ cells), KLN205 (2 × 10^4^ cells), H1975 (4 × 10^4^ cells), and SW900 (4 × 10^4^ cells) cells were seeded in 24 well plates and incubated for 24 h. The cells were treated with 0, 10, and 50 µM TAK242. After 24 h, apoptosis and necrosis were measured using the Annexin V-FITC and PI Apoptosis Detection Kit (Sigma Aldrich, Oakville, ON). The samples were analyzed using a flow cytometer (BD, Mississauga, ON). Apoptosis was defined as Annexin+ /Propidium iodide- (Ann+ /PI-) and necrosis/late apoptosis was defined as Ann–/PI+ and Ann+ /PI+ .

### 4.8 Colony formation assay

Five hundred cells of LLCI, KLN205, H1975, and SW900 were seeded per well in 24-well culture plates and treated with TAK-242, drug-containing media was removed after 48 h and drug-free media was added. The plates were further incubated until each colony consisted of about 50 cells (7−10 d). After fixation with 10% formalin, colonies were stained using crystal violet solution (0.5%w/v) and counted.

### 4.9 Clonogenic assay

Six-well plates were seeded with 100, 200, 400, 1000, 4000 LLCI, KLN205, SW900, and H1975 cells. Twenty-four hours post-incubation, the cells were treated with DMSO or TAK242 (10 and 25 µM) and exposed to different doses of irradiation corresponding to increasing cell densities (0, 2, 4, 6, or 8 Gy). Seven to ten days later, the cells were washed with PBS, fixed with formalin and stained with crystal violet. Colonies containing more than 50 cells were counted. The surviving fraction was calculated by dividing the number of colonies by the number of seeded cells and normalizing to the plating efficiency of the nonirradiated controls. The linear-quadratic model was used to fit the data.

### 4.10 Cell cycle analysis

NSCLC cells were treated with 25 µM TAK242 for 48 h to investigate the effects on the cell cycle distribution. Following treatment, the cell cycle was assessed using the Click-iT™ EdU Kit (Thermo Fisher Scientific, Montreal, QC). This method incorporates 5-ethynyl−2′-deoxyuridine (EdU), a thymidine analog, into newly synthesized DNA during the S phase of the cell cycle. After EdU incorporation, the cells were fixed and permeabilized, and the incorporated EdU was detected using a fluorescent azide-based reaction provided with the kit. The nuclear DNA was counterstained with DAPI (4',6-diamidino−2-phenylindole). Fluorescence signals for both EdU (FITC) and DNA content (DAPI) were analyzed by flow cytometry to determine the cell cycle phase distribution.

### 4.11 Statistical analysis

Statistical analysis was performed using GraphPad Prism 10.0.2 (GraphPad Software, Inc., La Jolla, CA). All experiments were performed in triplicate and repeated two to three times. One-way and two-way ANOVA analyzes or student's t-tests were performed for quantitative analyzes. A *P* value of <0.05 was considered significant.

## Data Availability

All data generated or analyzed in this study are fully available upon request from the corresponding author without restrictions.
